# Yiqihuoxuejiedu Formula Restrains Vascular Remodeling by Reducing the Inflammation Reaction and Cx43 Expression in the Adventitia after Balloon Injury

**DOI:** 10.1155/2015/904273

**Published:** 2015-10-19

**Authors:** Hong Chang, Huan Lei, Yizhou Zhao, Ruixue Yang, Aiming Wu, Yingqiu Mao, Youliang Huang, Xiying Lv, Jiuli Zhao, Lixia Lou, Dongmei Zhang, Yingkun He, Ying Xu, Tao Yang, Mingjing Zhao

**Affiliations:** ^1^Key Laboratory of Chinese Internal Medicine of Ministry of Education and Dongzhimen Hospital, Beijing University of Chinese Medicine, Beijing 100700, China; ^2^Beijing University of Chinese Medicine, Bei San Huan Dong Lu 11, Chao Yang District, Beijing 100029, China

## Abstract

Vascular remodeling is closely related to hypertension, atherosclerosis, and restenosis after PCI. Considerable evidence indicates that the activation and proliferation of adventitial fibroblasts play key roles in vessel injury. The inflammatory response and high expression of connexins contribute to adventitial remodeling. Therefore, reducing inflammation reaction and connexins expression in adventitia may become a new target to prevent vascular remodeling. Yiqihuoxuejiedu formula, composed of TCM therapeutic principle of supplementing qi, activating blood and detoxification, can inhibit restenosis after intimal injury. To further investigate the effect of Yiqihuoxuejiedu formula on inflammation and connexins, we established a carotid artery injury model. In model rats, hyperplasia in the intima was mild but obvious in the adventitia; CRP heightened; expressions of MCP-1, CD68, and Cx43 increased. Yiqihuoxuejiedu formula relieved intimal hyperplasia and adventitial area, obviously diminished the expressions of CD68 and Cx43 in the adventitia, and reduced CRP but did not lower MCP-1. These results indicated that Yiqihuoxuejiedu formula inhibited vascular remodeling especially adventitial hyperplasia by reducing the inflammation reaction including lowering macrophages infiltration and systemic nonspecific inflammatory response and also restraining gap junction connexins leading to less communication among cells. This study provides new ideas and methods for the prevention and treatment of vascular remodeling.

## 1. Introduction

Vascular remodeling is a structural and functional variation of vessels to adapt to the intracorporal environment. For a long time, vascular smooth muscle cells (VSMCs) in the media have been regarded as a central link and the adventitia has been known to play only supportive functions [1]. However, the adventitia is an essential regulator of vascular wall structure and function. Adventitial fibroblasts (AFs, the major component of the adventitia) are activated and transfer into myofibroblasts, proliferate, and migrate to media and intima to participate in the progression of vascular remodeling [2, 3].

In the initial stages of intimal balloon injury, one of the key triggers of vascular remodeling is early inflammation in the adventitia [4] including the infiltration of macrophages [5] and neutrophils [6] and the release of inflammatory factors, such as interleukin- (IL-) 1*β*, IL-6, IL-8, and MCP-1 [7, 8]. Research in patients also found that in-stent restenosis is related to macrophage infiltration [9]. Meanwhile myofibroblasts release various proinflammatory cytokines, for instance, MCP-1, recruiting macrophages and neutrophils to infiltrate into the adventitia [10, 11]. These inflammatory responses promote activation and proliferation of adventitial fibroblasts, resulting in adventitial remodeling.

Cellular interaction in blood vessels is maintained by multiple communication pathways, including gap junctions. Gap junctions (GJs) arise from the docking of two hemichannels or connexons, formed by the assembly of six connexins (Cxs), and achieve direct cellular communication by allowing the transport of small metabolites, second messengers, and ions between two adjacent cells [12]. Although Cx37, Cx40, and Cx43, respectively, are expressed in different layers of vessel wall, Cx43 is common in all the three layers [13–16]. Accumulating evidence supports the results that Cx43 has been deeply investigated in cardiovascular diseases. In atherosclerosis, Cx43 promoted leukocyte to adhere to endothelium and infiltrate into the media, which deteriorated atherosclerosis [17]. Further investigation showed that reduced Cx43 expression could inhibit atherosclerotic lesion formation in low-density lipoprotein receptor-deficient mice [18]. Meanwhile, upregulation of Cx43 promoted SMC phenotypic transformation and accelerated intimal hyperplasia, leading to vascular restenosis [19, 20]. In view of the function of Cx43, its expression and relationship with inflammation in the adventitia need to be illustrated.

In 2011 PCI guideline, treatment and prevention after PCI mainly include antihypertensive therapy including *β*-receptor blockers, angiotensin-converting enzyme inhibitors (ACEI), lipid-lowering therapy with statins, and antiplatelet/anticoagulant therapy with aspirin and clopidogrel [21]. Of these drugs, statins not only lower lipid concentration but also diminish inflammation and expression of GJ [22, 23]. Though western medicine has gained success, a number of potential risks remain, and we need to find new drugs to prevent vascular remodeling.

Myofibroblasts play pivotal role in the tissue repair and remodeling and are also a key player in pathological hypertrophic scars and organ fibrosis. The vascular remodeling after PCI is similar to local wound repair. Based on this idea, as well as the traditional Chinese medicine treatment of supplementing qi, activating blood plus detoxification, Yiqihuoxuejiedu formula was prescribed to prevent and treat vascular remodeling. The prescription is composed of astragalus, salvia, honeysuckle, and other components. Previous researches have shown that the prescription can reduce vessel stenosis, lower blood lipids [24], inhibit the activation and proliferation of the adventitial fibroblasts, and decrease collagen content and type I/III collagen ratio in the adventitia [25].

In this study, we used a vascular remodeling model of intimal injury with balloon injury and made Atorvastatin a positive control to explore the underlying mechanism of Yiqihuoxuejiedu's inhibition on adventitial inflammation and regulation of GJs at the early stages of injury (7 days). This study may provide a new approach to prevent vascular remodeling.

## 2. Materials and Methods

### 2.1. Animals

Male Sprague-Dawley (SD) rats weighing 380 to 420 g were purchased from Beijing Weitong Lihua Experimental Animal Technology Co., Ltd., Beijing, China (certificate number SCXK (Beijing) 2012-0001). Rats were raised in SPF room in Beijing University of Chinese Medicine. All the procedures were conformed to the National Institute of Health's Guide for the Use and Care of Laboratory Animals.

### 2.2. Establishment of Balloon Injury Model

One hour before anesthesia, a deep subcutaneous injection of heparin was given to each rat by 500 U/kg. Next, rats were anesthetized with 40 mg/kg sodium pentobarbital. As showed in the past study [25], a Fogarty 2 F balloon catheter (diameter of balloon 2 mm, length 20 mm, Medtronic Company, USA) was introduced through the left external carotid artery and advanced 4–4.5 cm into the thoracic aorta while the internal carotid artery was blocked. The balloon was inflated with normal saline at 0.5 atm to 0.7 atm to distend the artery. Then it was pulled back to the entry point. The entire procedure was repeated three times to denude the endothelium and cause vascular injury. After removing the catheter, the external carotid artery was ligated and the blood circulation of the internal carotid artery restored. In the sham group, only the external carotid artery was ligated. The above surgical operation was done in sterile condition.

### 2.3. Medications and Grouping

The Yiqihuoxuejiedu formula (Cat. Number 120603), composed of astragalus, salvia, honeysuckle, and other components, was produced by the Chinese Herbal Company of Beijing University of Chinese Medicine (Beijing, China) and the final concentration of crude drug was 1.2 g/mL.

Rats with balloon injury were randomly divided into three groups: the model group, the Atorvastatin group, and the Yiqihuoxuejiedu group. The sham group served as a control. Rats in the Atorvastatin group were orally administered with 13.33 mg/kg/d of Atorvastatin calcium (Pfizer), while they were administered with 12 g/kg in the Yiqihuoxuejiedu group and 10 mL/kg/d distilled water in the sham and model groups. Both doses for the two groups were based on the typical daily clinical dosages for adults, corresponding with 10 times of clinical dosages. Rats were administered once a day and seven days later, perfused with 4% paraformaldehyde through the left ventricle to fix specimen and make paraffin sections.

### 2.4. Histomorphometric Analysis

Carotid artery sections (5 *μ*m) were stained with hematoxylin-eosin. Next, the sections were examined with a microscope (magnification ×100) and photographed for morphological analysis. Image analysis software (Image-Pro Plus 6.0) was used to analyze the following morphological indicators: lumen radius (lumen perimeter/2*π*), neointimal thickness (internal elastic membrane perimeter/2*π* − lumen perimeter/2*π*), and adventitial area (total area of vessel − area circled by external elastic membrane).

### 2.5. Radioimmunoassay Measurement and Analysis of CRP

For radioimmunoassay of CRP, a nonequilibrium method was used. The corresponding antibody was used to the standard and sample solutions, mixed well, and placed at 4°C for 24 h. Next, 125I-VIP (125I-SP, 125I-SS, Beijing Huaying Biotechnology Institute) was added, mixed well, and placed at 4°C for another 24 h. After that, appropriate secondary antibody was added, mixed thoroughly, and placed at room temperature for 20 min. Then samples were centrifuged 3500 r for 20 min, and the supernatants were discarded. The radionuclide blink count of precipitation was tested and converted to mg/L according to the standard curve.

### 2.6. Immunohistochemistry Measurement and Analysis of MCP-1, CD68, and Cx43

Different antibodies were used to detect the expressions of MCP-1, CD68, and Cx43 in the vascular wall. All samples were repaired in a microwave and incubated in 0.3% hydrogen peroxide before using primary antibodies (MCP-1, Abcam, UK; CD68 and Cx43, Santa Cruz Biotechnology, USA) and secondary antibody-HRP multimer (Zhongshan Golden Bridge Biotechnology Company, China). Samples were then visualized with diaminobenzidine substrate. The positive expressions of CD68, Cx43, and MCP-1 appeared as brownish-yellow or a brown granulation in the cytoplasm. The typical images were captured with SPOT V3.0II software, and the average optical density or the positive area was measured and analyzed by image-Pro Plus 6.0.

### 2.7. Statistical Analysis

Mean differences among groups were statistically analyzed using one-way analysis of variance (ANOVA) and between two groups by a TSD test. The level of statistical significance was considered to be *P* < 0.05.

## 3. Results

### 3.1. Lumen Radius and Changes of Neointimal Thickness

**Figure 1:**
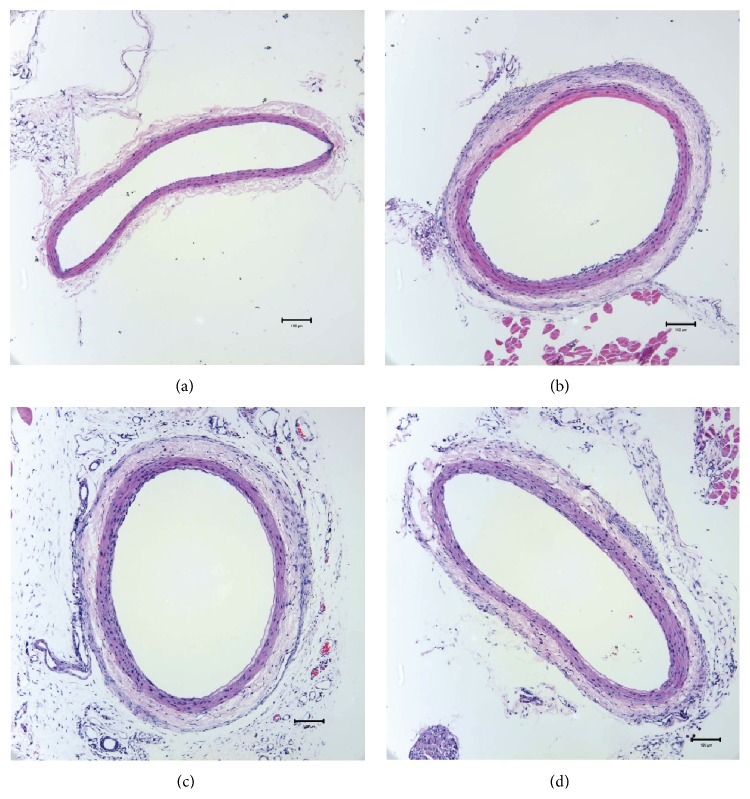
Left common carotid artery slices with HE 7 days after injury. (a) Sham group, (b) model group, (c) Atorvastatin group, and (d) Yiqihuoxuejiedu group.

**Figure 2:**
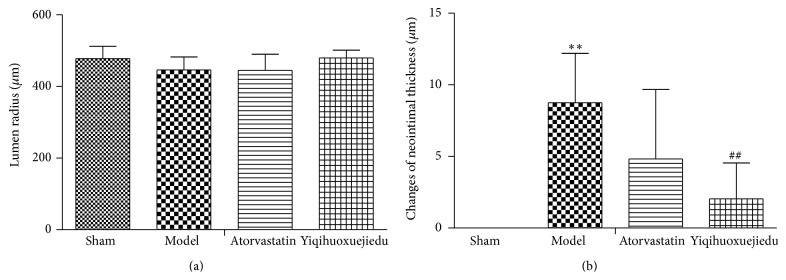
Lumen radius and changes of neointimal thickness 7 days after balloon injury. (a) Lumen radius. (b) Changes of neointimal thickness. ^*∗∗*^Compared with sham group, *P* < 0.01. ^##^Compared with model group, *P* < 0.01.

Seven days after balloon injury, there was small reduction of lumen radius in the model group but had no significant difference compared with the sham group, neither in Atorvastatin nor in Yiqihuoxuejiedu groups. Intimal hyperplasia appeared obviously in the model group compared to the sham group (*P* < 0.01). Compared with the model group, the Yiqihuoxuejiedu formula could reduce neointimal thickness (*P* < 0.01, Figures 1 and 2).

### 3.2. The Area of the Adventitia

**Figure 3:**
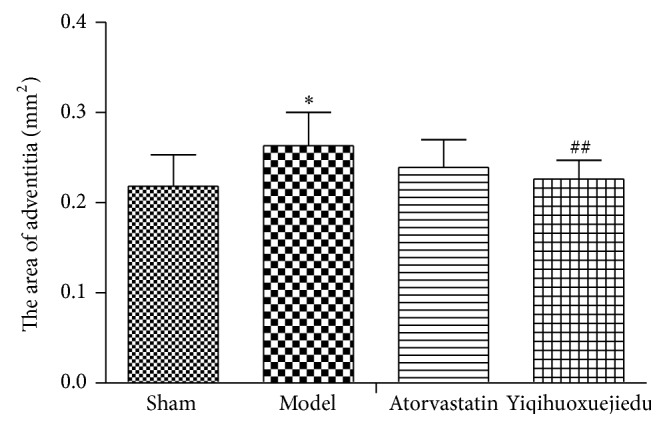
The area of adventitia 7 days after balloon injury. ^*∗*^Compared with sham group, *P* < 0.05. ^##^Compared with model group, *P* < 0.01.

There was a significant increase in the area of the adventitia in model group (*P* < 0.05). Compared with the model group, the area of the adventitia in the Yiqihuoxuejiedu group was decreased (*P* < 0.01, Figures 1 and 3).

### 3.3. The Concentration of CRP in Serum

**Figure 4:**
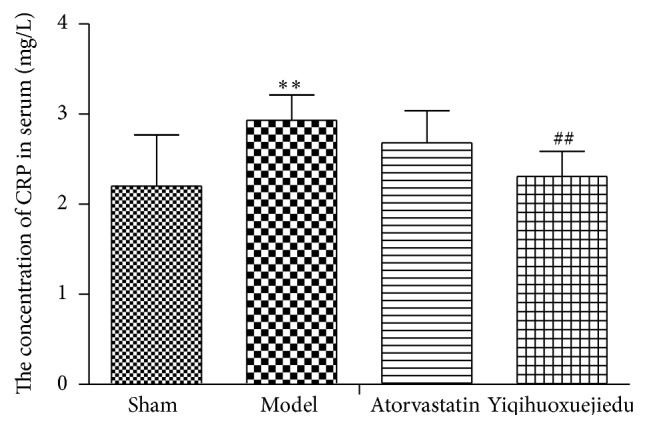
The concentration of CRP in serum. ^*∗∗*^Compared with sham group, *P* < 0.01. ^##^Compared with model group, *P* < 0.01.

At the early period of injury, CRP increased markedly in the serum, especially in the model group (*P* < 0.01). The Yiqihuoxuejiedu formula decreased CRP (*P* < 0.01), while Atorvastatin only had a trend in reducing CRP (Figure 4).

### 3.4. The Expression of MCP-1 in Vascular Wall

**Figure 5:**
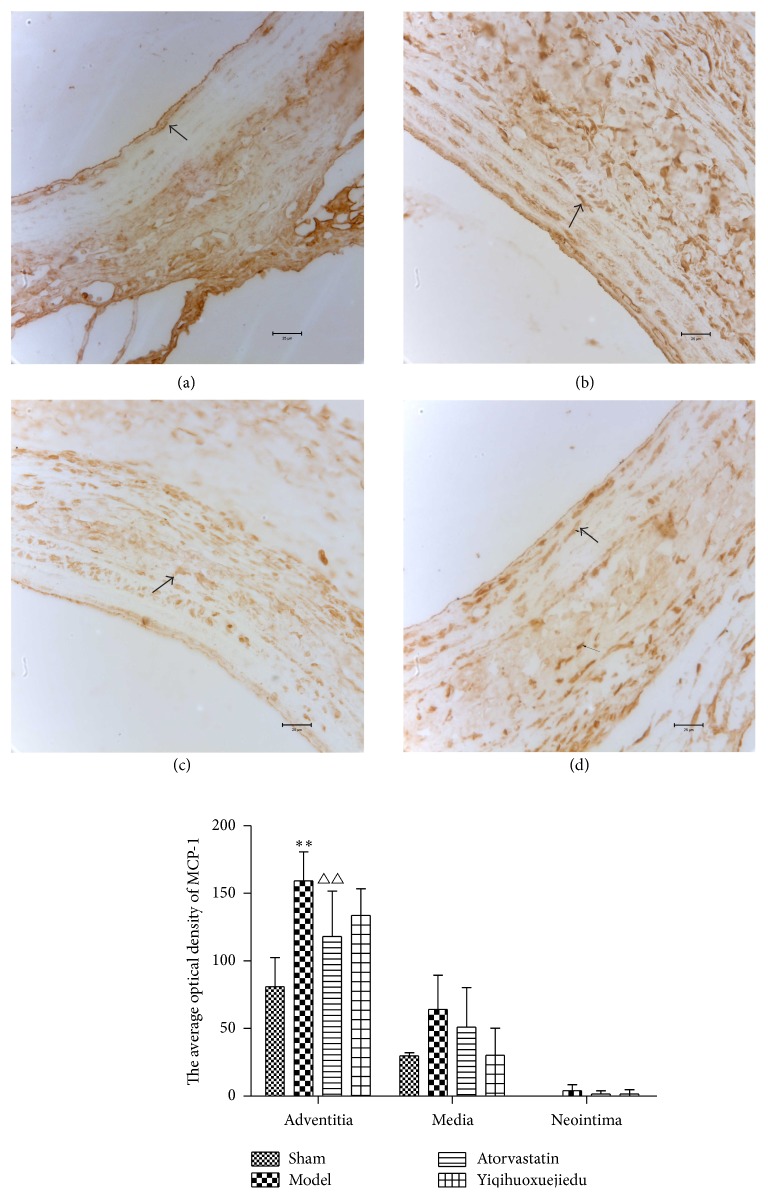
The expression of MCP-1 in vascular wall. (a) Sham group, (b) model group, (c) Atorvastatin group, and (d) Yiqihuoxuejiedu group. ^*∗∗*^Compared with sham group, *P* < 0.01. ^##^Compared with model group, *P* < 0.01.

Immunohistochemistry showed that the expression of MCP-1 in the three layers of vascular wall increased after balloon injury, especially in the adventitia of the model group. The average optical density (OD) in the treatment groups was all decreased; the adventitial positive expression in the Atorvastatin group had an apparent reduction compared with the model group (*P* < 0.01, Figure 5).

### 3.5. The Expression of CD68 in Vascular Wall

**Figure 6:**
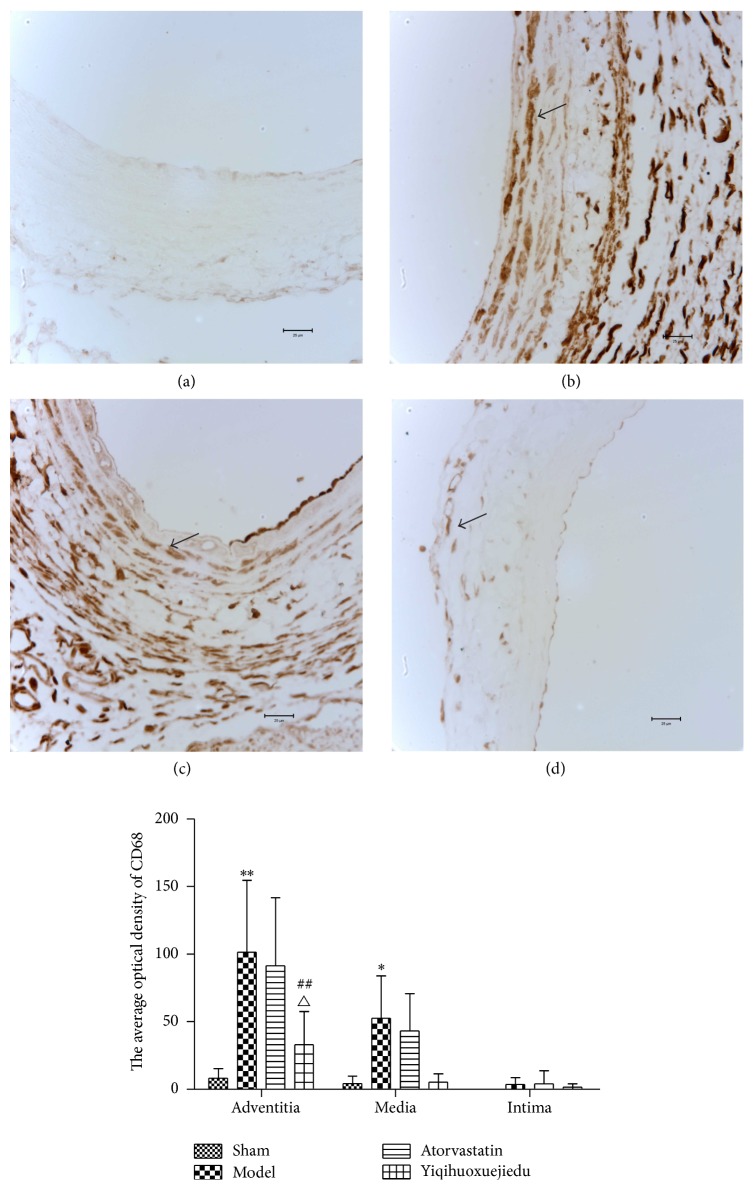
The expression of CD68 in vascular wall. (a) Sham group, (b) model group, (c) Atorvastatin group, and (d) Yiqihuoxuejiedu group. ^*∗∗*^Compared with sham group, *P* < 0.01. ^##^Compared with model group, *P* < 0.01. ^△^Compared with Atorvastatin group, *P* < 0.05.

Compared with the sham group, CD68 expression of media and adventitia in the model group had a significant increase (*P* < 0.05 for media, *P* < 0.01 for adventitia). The Yiqihuoxuejiedu formula inhibited positive expression of CD68 in the adventitia (*P* < 0.01), and it had a stronger effect than Atorvastatin (*P* < 0.05, Figure 6).

### 3.6. The Expression of Cx43 in Vascular Wall

**Figure 7:**
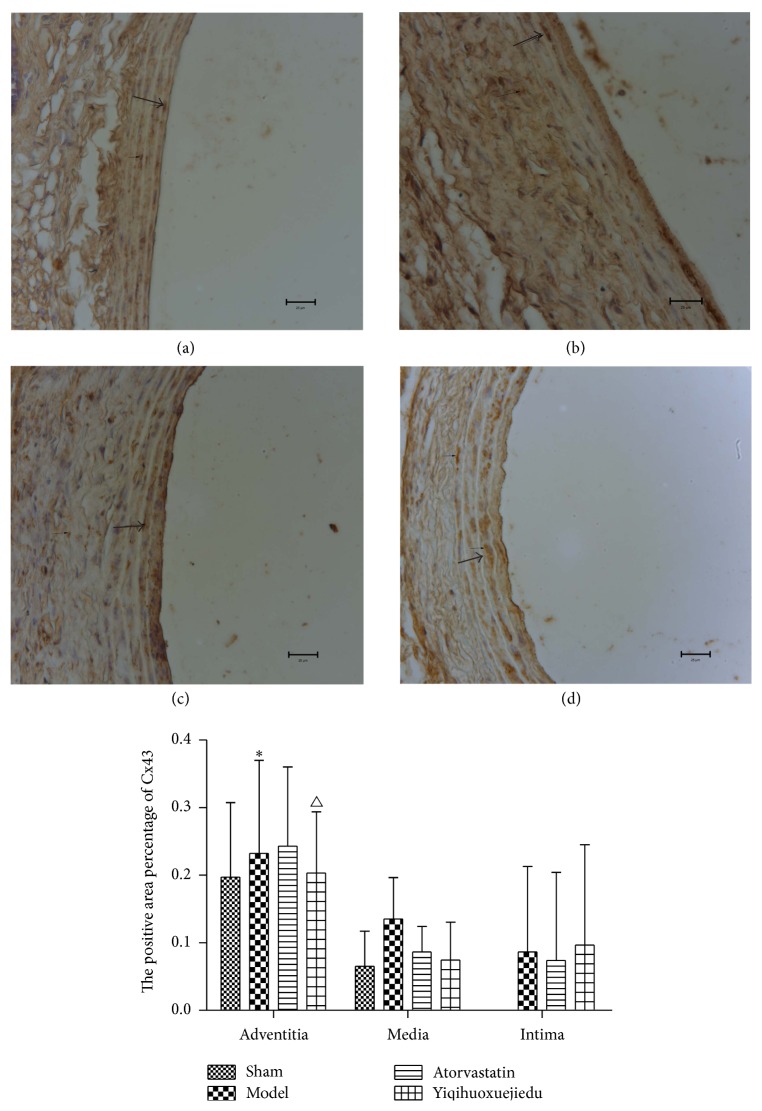
The expression of Cx43 in vascular wall. (a) Sham group, (b) model group, (c) Atorvastatin group, and (d) Yiqihuoxuejiedu group. ^*∗*^Compared with sham group, *P* < 0.05. ^#^Compared with model group, *P* < 0.05.

There were no significant changes in the average OD in the vascular wall when determining Cx43 expression. However, the positive areas of Cx43 in surgery groups were increased, and there was significant difference between the model group and the sham group (*P* < 0.05). Compared with model group, the positive area of Cx43 in adventitia significantly decreased in the Yiqihuoxuejiedu group (*P* < 0.05). We observed the same trend in the media but without any significant differences (Figure 7).

## 4. Discussion

PCI can lead to two types of vascular remodeling, positive remodeling and negative remodeling [26]. Positive remodeling means that the vessels provide compensatory dilation, and the lumen diameter does not change significantly, while negative remodeling means that vessels shrink, and the lumen diameter narrows. In the early remodeling, adventitial fibroblasts play principal roles, although VSMCs also contribute to the pathological change. In this study, neointimal thickness slowly increased and lumen showed no stenosis, but the adventitial area rapidly grew in model animals. Thus, positive remodeling dominated vascular remodeling at the early phase of intimal injury. The Yiqihuoxuejiedu formula could inhibit neointimal thickness and reduce the adventitial area. Our previous study demonstrated that *α*-SMA expression of the adventitia increased in model group at 7 days after balloon injury, which indicated activation and proliferation of the adventitial fibroblasts [25].

C-reactive protein (CRP) not only serves as one of the most widely known biomarkers of cardiovascular disease and underlying inflammation but also promotes activation of endothelial cells and monocytes, leading to vascular remodeling [27, 28]. The concentration of CRP increases when inflammation occurs in the adventitia [8]. In our study, CRP increased highly in serum, particularly in the model group (*P* < 0.01), while the Yiqihuoxuejiedu formula could diminish CRP (*P* < 0.01).

Lesion of the intima can lead to an early inflammatory response, including infiltration of macrophages and neutrophils and the release of inflammatory factors, especially in the adventitia [29]. MCP-1 is secreted by monocytes-macrophages and adventitial fibroblasts [30, 31], and it can stimulate VSMCs to proliferate and migrate [32, 33] and promote monocytes to gather in the adventitia, leading to AFs proliferation and adventitial thickness [7]. In our study, MCP-1 expression increased in all the three layers of the vessel wall and markedly increased in the adventitia (*P* < 0.01). The concentration of MCP-1 was higher in the adventitia than in the media and neointima, indicating severe adventitial inflammation after vascular injury. The positive medicine, Atorvastatin, significantly reduced MCP-1 expression to restore the body's balance between proinflammatory and anti-inflammatory responses [34], but the Yiqihuoxuejiedu formula had no effect on MCP-1.

Thirty minutes after intimal injury, the inflammatory cells can be detected in the adventitia, with earlier time and higher concentration than in the media [6]. Macrophages can release cytokines and promote neointimal hyperplasia [35–37]. CD68 is a special marker of macrophages. Our study showed that macrophages infiltrated into the media and adventitia, particularly in the latter. The Yiqihuoxuejiedu formula limited the infiltration of macrophages dramatically, and its effect was better than that of Atorvastatin (*P* < 0.05), while Atorvastatin failed to do this.

GJs are important channels for exchanging matter, energy, and information between cells. They are closely associated with vascular function. Connexins (Cxs) are the main components of GJs, and Cx43 is expressed mostly in the vascular wall [16, 38]. Previous studies have indicated that Cx43 in vessel wall increased after PCI and played a promoting role in vascular remodeling by encouraging VSMCs to proliferate and be activated together, leading to inflammation in injured vessels [39–41]. One of the drug targets is to reduce the level of Cxs and communication among cells to prevent and treat remodeling. The present study showed that Cx43 expression increased in the vessel wall, especially in the adventitia, and cell communication strengthened, leading to cell proliferation and inflammation. Yiqihuoxuejiedu formula significantly reduced Cx43 (*P* < 0.05). Atorvastatin downregulated Cx43, but the change was not significant. Other studies found different results with Atorvastatin, mostly showing that the drug caused limited Cx43 expression in VSMCs to achieve an antiproliferative role [23]. Different drug doses may account for the different results. We used 10 times the clinical dose in our study, but other studies used higher doses [42, 43]. Thus, it is unclear whether stains have an effective role in GJs under clinical doses.

Yiqihuoxuejiedu formula is composed of astragalus, salvia, honeysuckle, and other components based on Chinese medicine principle of strengthening qi, activating the blood plus detoxication. Our previous studies have demonstrated that this formula reduced levels of lipids and TGF-*β* [24], limited neointimal hyperplasia, and diminished the collagen deposition in neointimal formation [44]. Astragalus, salvia, and tetramethylpyrazine, the main ingredients, play effective roles in anti-inflammation, downregulating MCP-1 and inhibiting SMC proliferation and migration [45–50]. All of these targets are the vital pharmacological bases for the Yiqihuoxuejiedu formula to inhibit neointimal formation and reduce macrophages infiltration and Cx43 expression in present study.

## 5. Conclusion

In summary, the injured vessel presents positive remodeling with slight neointimal hyperplasia and remarkable adventitial remodeling, and it also elicits inflammatory reactions with high expressions of MCP-1, CD68, and Cx43, particularly in the adventitia at the early phase of intimal injury. Yiqihuoxuejiedu formula restrained expression of CD68 and Cx43 remarkably in the adventitia and reduced the concentration of CRP and diminished intimal hyperplasia and area of the adventitia. These results indicated that this formula could inhibit vascular remodeling by limiting macrophages infiltration in the adventitia, depressing systemic nonspecific inflammatory response and AFs proliferation, and reducing GJs between cells. This study provides new ideas and methods for the prevention and treatment of vascular remodeling.
